# Supplementary material for: Projected decrease in trail access in the Arctic
Source: Commun Earth Environ. 2023 Feb 3;4(1):23. doi: 10.1038/s43247-023-00685-w (PMC11041733; doi:10.1038/s43247-023-00685-w)
Supplement: Supplementary file 1 — Supplementary Information [file 43247_2023_685_MOESM1_ESM.pdf]

Supplementary materials: Projected decrease in trail access in the Arctic

[1]Ford, J.D., [2]Clark, D.G., [3]Copland, L., [4]Pearce, T., [5]IHACC Research Team*, [6]Harper, S.L.

1. Supplementary methods

Steps 1: Semi-structured interviews

As described in Ford et al (1), 273 semi-structured interviews were conducted with regular trail users in 9 communities, with the aim of developing a generalizable understanding of climate-relevant conditions affecting trail access across Inuit Nunangat. Interview questions focused on documenting knowledge about past and current use of trails, the nature of climate-related conditions posing risks, and how risks are perceived and managed. More can be found about this step in Ford et al (1).

Step 2: Trail use thresholds

Trail use thresholds were created by translating qualitative data (Step 1) into thresholds for climate and sea ice conditions. This involved developing a list of variables specific to each climate-related condition that could be measured, which define whether a trail can be used or not, focusing on three trail modes. To account for variation in knowledge, skills sets, equipment, and risk tolerance of trail users, thresholds were set differently for different categories of trail user: Type 1 (normal risk tolerance); Type 2 (low risk tolerance); and Type 3 (high risk tolerance).

Supplementary Table S1: *Trail user types*

	**Type 1: Normal risk tolerance**	**Type 2: Low risk tolerance**	**Type 3: High risk tolerance**
**Access to quality and appropriate equipment**	Average	Below average quality	High quality equipment
**Inuit traditional knowledge**	Average	Below average	High level of Inuit knowledge
**Risk tolerance**	Average	Lower risk tolerance	Higher risk tolerance

To account for the change from point-based data used in Ford et al (1) to gridded climate data, we had to calibrate the thresholds. We began Step 2 by using the trail thresholds described in Ford et. al. We then converted the thresholds unit values as required to match climate modeling standards (i.e. Celsius is used in Ford et al (1) and climate models use Kelvin). Next, after running the models (Step 3 to Step 5), we adjusted thresholds so that historic estimates for the 2010-2016 period (data from Ford et al (1)) were within +- 30% of the projected 2015 to 2020 values for each region and user type. We were able to determine which specific threshold needed to be adjusted by examining the average number of 'fails' by variable in the models. The final thresholds used for the models are listed in Supplementary Table S1

Supplementary Table S2: *Model thresholds*

**Trail Mode**	**User Type**	**Variable**	**Variable unit**	**Low threshold** *(daily projection below x is a 'fail)*	**High threshold** *(daily projection above x is a 'fail)*	**Importance to Inuit** [1]	**Insights for threshold development** [1]	**Literature**
Land	Average skill (Type 1)	Temp	Celsius	5	-5	• Temperatures around freezing are linked to muddy/slushy conditions that are difficult to drive an ATV or snowmobile in<br>• Rocks are more exposed during spring and fall, making travel difficult<br>• Snowmobiles are more likely to overheat during warmer weather	Temperatures between -5 and 5°C have been statistically associated with increased risk of injury	[2,3]
	Low skill (Type 2)	Temp	Celsius	6	-6			
	High skill Type 3)	Temp	Celsius	4	0			
	Average skill (Type 1)	PrcpRain	kg m^{-2} s^{-1}		1.16x10-04	• Rain is generally not desired by travellers<br>• If temperatures are just above freezing, precipitation can pose a travel risk<br>• White-out (very low visibility due to blowing snow) conditions are not favourable	• 5mm of daily rainfall can equal 10cm (1.16x10-3 kg m^{-2} s^{-1}) of snow in the winter, which can result in risky travel conditions. Similarly, more than 1cm of rain (1.16x10-4 kg m^{-2} s^{-1}) would be uncomfortable and risky.<br>• *Sallivaliajuq* refers to ice thinning due to rain or wind.	[4]
	Low skill (Type 2)	PrcpRain	kg m^{-2} s^{-1}		5.79x10-05			
	High skill Type 3)	PrcpRain	kg m^{-2} s^{-1}		1.73x10-04			
	Average skill (Type 1)	PrcpSnow	kg m^{-2} s^{-1}		5.79x10-05			
	Low skill (Type 2)	PrcpSnow	kg m^{-2} s^{-1}		2.31x10-05			
	High skill Type 3)	PrcpSnow	kg m^{-2} s^{-1}		1.16x10-04			
	Average skill (Type 1)	WndWrm	m s^{-1}		11.11	• Wind during the winter can create ground blizzards and limit visibility	Wind > 30km/hr (8.33 m s^{-1}) is uncomfortable and has potential to be unsafe	[5,6]
	Low skill (Type 2)	WndWrm	m s^{-1}		8.33			
	High skill Type 3)	WndWrm	m s^{-1}		13.89			
	Average skill (Type 1)	WndCld	m s^{-1}		5.56			
	Low skill (Type 2)	WndCld	m s^{-1}		4.17			

	High skill Type 3)	WndCld	m s^{-1}		9.72	• Wind during winter can create unfavourably cold conditions<br>• Wind in the summer is less of a travel challenge and is generally just viewed as not desirable		
	Average skill (Type 1)	Vis	km	1		• Poor visibility can make navigation more difficult and slows travel speed	> 1km is preferred by most travellers	[7]
	Low skill (Type 2)	Vis	km	2				
	High skill Type 3)	Vis	km	1				
Ice	Average skill (Type 1)	Temp	Celsius		0	• Temperatures near and above freezing can create instability	Temperatures between -5 and 5°C have been statistically associated with increased travel risk	[1]
	Low skill (Type 2)	Temp	Celsius		-5			
	High skill Type 3)	Temp	Celsius		5			
	Average skill (Type 1)	Prcp	kg m^{-2} s^{-1}		3.47x10-05	• Snow is generally associated with poor visibility<br>• Snow can limit ability to see ice colour and read ice thickness<br>• Rain can cause ice quality and safety to diminish rapidly	It was considered to be less safe on the ice during precipitation than on land. 3mm of rain (3.47x10-5 kg m^{-2} s^{-1}) or 9cm of snow in a 24-hour period would likely create unsafe conditions	[4]
	Low skill (Type 2)	Prcp	kg m^{-2} s^{-1}		1.16x10-05			
	High skill Type 3)	Prcp	kg m^{-2} s^{-1}		5.79x10-05			
	Average skill (Type 1)	Wnd	m s^{-1}		8.33	• Ice break up happens quicker with high winds<br>• Wind can increase risk as leads open up<br>• Waves can cause air pockets under the ice near the floe edge	Depending on the location, wind can have more or less of an impact on safety; generally, >30km (8.33 m s^{-1}) creates safety issues	[4,6,10,11]
	Low skill (Type 2)	Wnd	m s^{-1}		4.17			
	High skill Type 3)	Wnd	m s^{-1}		11.11			
	Average skill (Type 1)	Vis	km	1.5		• Low visibility can slow travel and make it difficult to see leads in the ice	Specific cut-off is location dependant; however, 2km is generally considered the minimum	[4]
	Low skill (Type 2)	Vis	km	3				
	High skill Type 3)	Vis	km	2.5				

	Average skill (Type 1)	IceConc	% area covered	45		• Low ice concentrations can make it difficult to travel across the ice<br>• Ice thickness to hold snowmobile and qamitik is necessary	Local areas of 80% concentration is generally assumed to be navigable, which regionally is roughly equal to 45% concentration	6,8-10,12-14
	Low skill (Type 2)	IceConc	% area covered	50				
	High skill Type 3)	IceConc	% area covered	40				
	Average skill (Type 1)	IceThick	meters	0.35				
	Low skill (Type 2)	IceThick	meters	0.4				
	High skill Type 3)	IceThick	meters	0.25				
Water	Average skill (Type 1)	Temp	Celsius	-5		• Temperatures around and below zero are not generally desired for travel; however, some types of harvesting may benefit<br>• Temperatures below -10°C generally mean ice is forming and that it may be more difficult to return to the floe edge or shore	Temperatures below -5°C are thought to cause discomfort or elevated travel risk on the water	4
	Low skill (Type 2)	Temp	Celsius	0				
	High skill Type 3)	Temp	Celsius	-13				
	Average skill (Type 1)	Prcp	kg m^{-2} s^{-1}		4.63x10-05	• Rain and snow is generally not desired, but if it does not decrease visibility, it is generally safe	Greater than 5mm (5.79x10-5 kg m^{-2} s^{-1}) of precipitation in a day would likely limit comfort and visibility	
	Low skill (Type 2)	Prcp	kg m^{-2} s^{-1}		1.16x10-05			
	High skill Type 3)	Prcp	kg m^{-2} s^{-1}		9.26x10-05			
	Average skill (Type 1)	Wnd	m s^{-1}		5.56	• Wind can blow ice back to floe edge or shore and limit ability to return<br>• Most vessels used are not capable of handling medium and large waves	Wind over 35km/hr (9.7 m s^{-1}) has potential to create waves near 1m, which is beyond most private boat capacities	4,5
	Low skill (Type 2)	Wnd	m s^{-1}		4.17			
	High skill Type 3)	Wnd	m s^{-1}		8.33			
	Average skill (Type 1)	Vis	km	2.5		• Good visibility is needed to detect changes in nearby ice conditions	Literature and individual perspectives vary, but generally visibility of at least >6km is required for safe travel	4
	Low skill (Type 2)	Vis	km	4				
	High skill Type 3)	Vis	km	1				

						• Good visibility is important for ease of navigation when there is no GPS available		
	Average skill (Type 1)	IceConc	% area covered		25	• Low or no ice concentrations are preferred<br>• Ice thickness of any amount detectable is not favourable for travel	Less than 30% ice coverage generally preferred locally. At a regional scale this equates to roughly 25% coverage	[4,6,8,9]
	Low skill (Type 2)	IceConc	% area covered		20			
	High skill Type 3)	IceConc	% area covered		40			

Step 3: Trail access models

We developed a script in R Cran that iteratively assessed each the ‘pass’ and ‘fail’ criteria using a set of nested IF / THEN statements. The model was built to work through daily timeseries data and pull threshold values from an independent table.

Step 4: Visibility timeseries

Ground-level visibility is not projected by global climate models under CMIP6. However, visibility is described as being an important weather condition that can constrain travel and was used in the historic analysis (1). In order to capture visibility in our analysis, we developed a synthetic timeseries by randomly sampling historic daily visibility data from across Inuit Nunangat.

Daily ground-level visibility data was pulled from Environment Canada’s historic weather database. All weather stations data from locations within the study area were examined to determine which stations had longitudinal data from the period of 2000 to 2020. Once relevant stations were identified, daily data was downloaded and grouped by region (Inuvialuit Settlement Region (ISR), Ellesmere, Baffin, Kitikmeot, Kivalliq, Nunavik, and Nunatsiavut). We then created a table of daily visibility values for each region and, using R Cran, randomly sampled each region’s data 31,390 times (86 years * 365 days). This yielded a timeseries that was equivalent in length to the timeseries of climate data (Step 5) and which had a distribution nearly the same as historic observations. As evident in the supplementary table below, visibility observational data is not consistently collected in many locations across Inuit Nunangat. Stations with longitudinal visibility data used for our synthetic timeseries are captured in Supplementary Table S2.

Supplementary Table S3: *Stations with longitudinal visibility data*

Region	Station Names
Baffin	Arctic Bay; Cape Dorset Automated Weather Observation System (AWOS); Clyde AWOS; Hall Beach AWOS; Iqaluit A; Pond Inlet AWOS; Qikiqtarjuaq AWOS
Ellesmere	Grise Fiord A; Resolute Bay A
Inuvialuit Settlement Region	Tuktoyaktuk A;
Kitikmeot	Paulatuk A; Gjoa Haven AWOS; Kugluktuk A; Lupin A; Taloyoak A
Kivalliq	Arviat AWOS; Baker Lake A; Coral Harbour A; Rankin Inlet A
Nunavik	Kuujjuarapik A; Kuujjuaq A
Nunatsiavut	Makkovik A;

Step 5: Climate and ice projections

Our modeling uses climate data from CMIP6 global climate models. At the time of the analysis, there were no statistically downscaled data from CMIP6 available for Canada. Further, CMIP5 data could not be used since daily data for sea ice concentration and sea ice thickness were not statistically downscaled for Canada. We used all GCMs that offered all required climate variables at 100km spatial resolution (see Supplementary Table S3)

Data for each GCM, emissions scenario (SSP 245 and 585), and variable (tas, pr, sfcWind, siconc, sithick) were downloaded from a CMIP6 climate data portal (https://pcmdi.llnl.gov/CMIP6/) using R Cran. Data was downloaded in NetCDF format for the time period of 2015 to 2100.

Next, we developed two spatial polygons to capture the study area for both climate variables and sea ice variables. Buffer areas of 100km radius were used for climate data (tas, pr, and sfcWind) while a buffer of 150km radius was used for sea ice data. The difference in the buffer size reflected the need to capture an adequate sample size of grid cells and was informed by our understanding of trail networks across Inuit Nunangat. Buffers around all Inuit Nunangat communities were created using ESRI ArcGIS.

We used R Cran to develop point-based timeseries for each region. First, each gridded climate projection (NetCDF format) was cut to each regional polygon. The mean value of all gridded cells within each polygon was then calculated for each day, GCM, emissions scenario, and variable. These data were passed and collated to a database of all projection data for each GCM. The script for pulling and aggregating climate data used the following packages in R Cran: epwshiftr; RNetCDF; raster; ncdf4; chron; lattice; hdf5r; ncdf4.helpers; PCICt; sf; rgeos; and svMisc

Finally, we created an ensemble timeseries based on the mean value of climate and ice variables for each GCM, emissions scenario, and region.

Supplementary Table S4: *We used all GCMs that offered all required climate variables at 100km spatial resolution (selected GCMs in green). Data captured January 2022.*

Model Organization	Model	sfcWind	siconc	sithick
AS.RCEC	TaiESM1	√		
AWI	AWI-CM-1-1-MR	√		
BCC	BCC-CSM2-MR	√	√	√
CCCma	CanESM5		√	
CMCC	CMCC-ESM2	√	√	√
CMCC	CMCC-CM2-SR5	√	√	√
DKRZ	MPI-ESM1-2-HR	√		
EC-Earch-Consortium	EC-Earth3	√	√	
EC-Earch-Consortium	EC-Earth3-CC		√	√
EC-Earch-Consortium	EC-Earth3-Veg		√	
EC-Earch-Consortium	EC-Earth3-Veg-LR		√	
INM	INM-CM4-8	√		
INM	INM-CM5-0	√		
IPSL	IPSL-CM6A-LR		√	√
MIROC	MIROC6		√	√
MRI	MRI-ESM2-0	√	√	√
NCC	NorESM2		√	
NCC	NorESM2-LM		√	√
NCC	NorESM2-MM	√	√	√
NOAA-GFDL	GFDL.ESM4	√		
NOAA-GFDL	GFDL-CM4	√		
NUIST	NESM3		√	√

Figure S1: *Gridded climate model cells that were selected for sea ice projections. Cells were selected within 150 km of communities in Inuit Nunangat. GCMs did not have data for all cells; NAs were removed before the mean for each region was calculated. Each region is noted in this map with a label and unique color.*

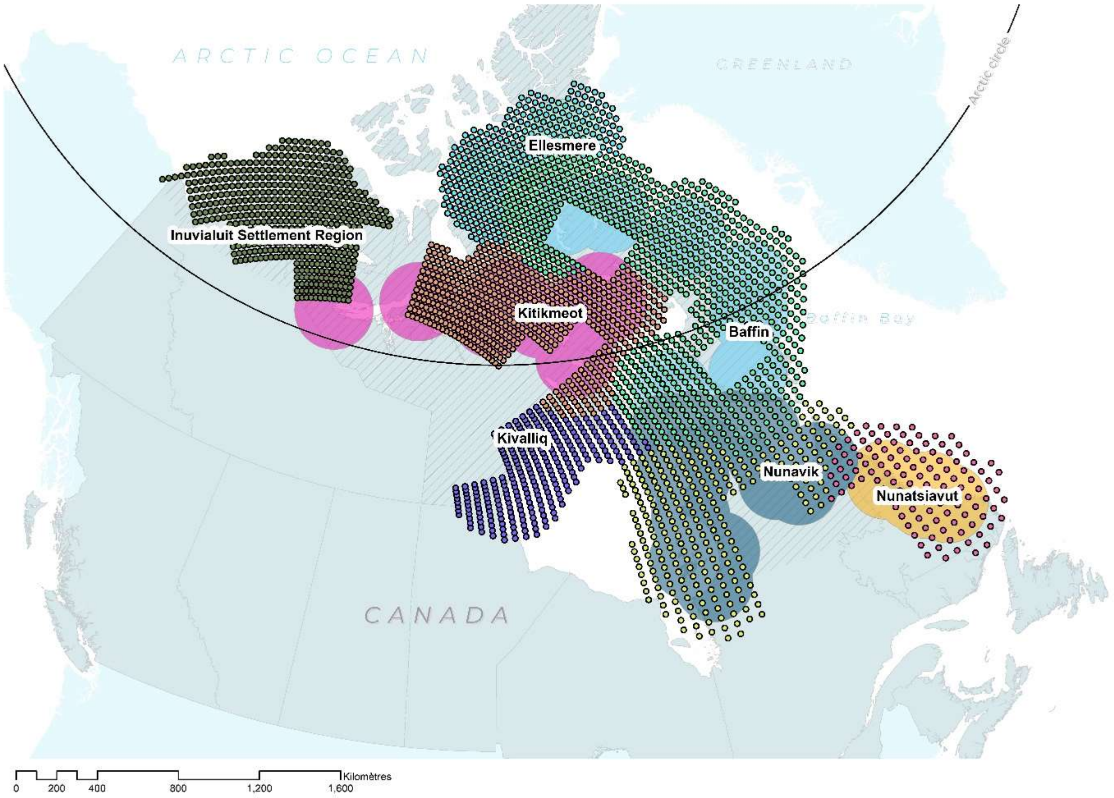

Step 6: 'Pass' and 'fail' days

For each GCM and emissions scenario, we calculated 'pass' and 'fail' days by applying the models from Step 3 to the timeseries of climate and ice projections as well as the synthetic visibility timeseries. We ran the 'pass' and 'fail' analysis using R Cran.

Step 7: Analysis

For the analysis, we aggregated findings to annual averages. We also examined decadal distributions of daily data.

We deemed a day as a 'good trail day' if there were no fails on the given day.

We determined 'trail access days' or days when at least one mode of trail was available, by examining daily land, ice, and water projections.

2. Supplementary results

Variations in temperature and sea ice projections by region

Figure S2: *Projected changes in temperature and sea ice for SSP245 and 585.*

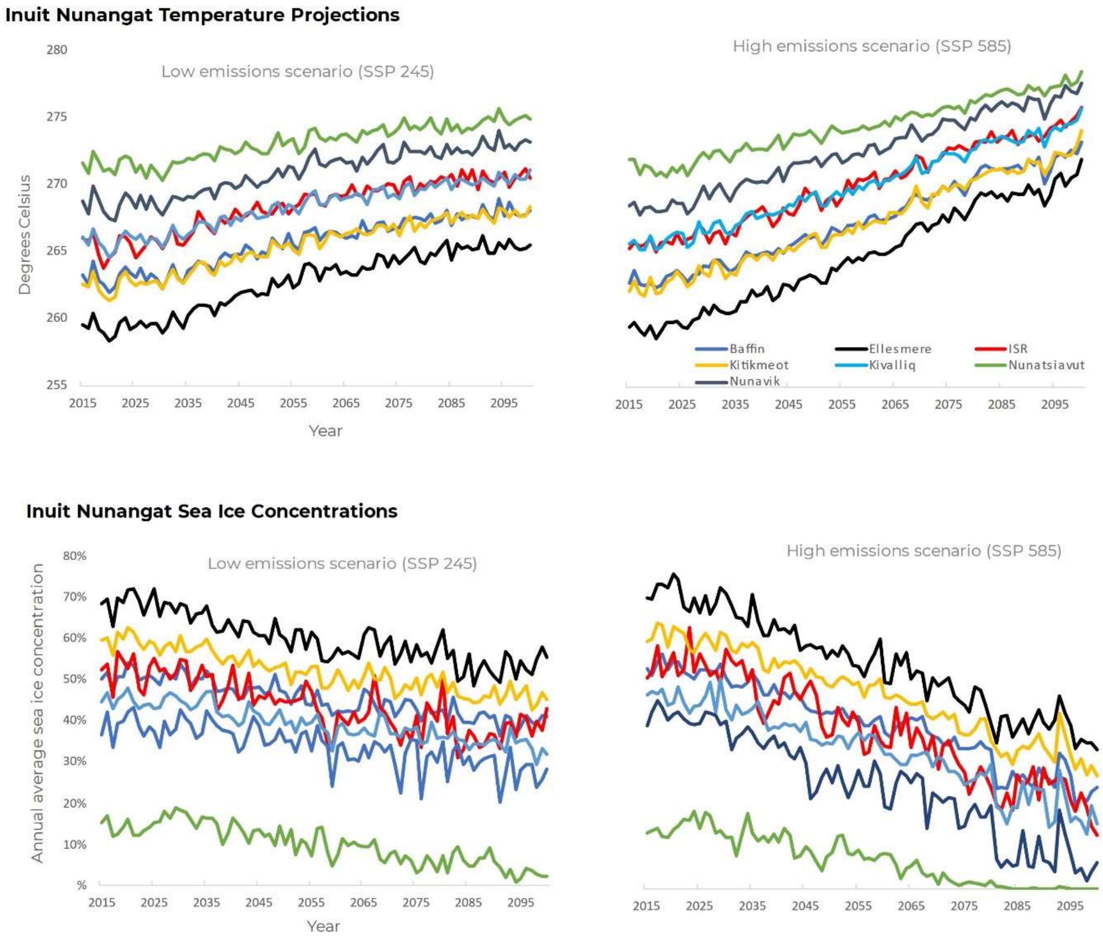

Projected precipitation changes

The largest percent change precipitation is projected under the high emission scenario and for the higher latitude regions (Fig. S1). Models project annual average precipitation in Baffin, for example, will increase by 43% compared to 2010 the end of the century. However, even under the low emissions scenario, models project an 8% increase in annual precipitation compared to 2010 in Nunatsiavut by the end of the century. Precipitation was the least common climate or ice variable to cause a model 'fail' across Inuit Nunangat.

Figure S3: *Projected changes in precipitation for SSP245 and 585.*

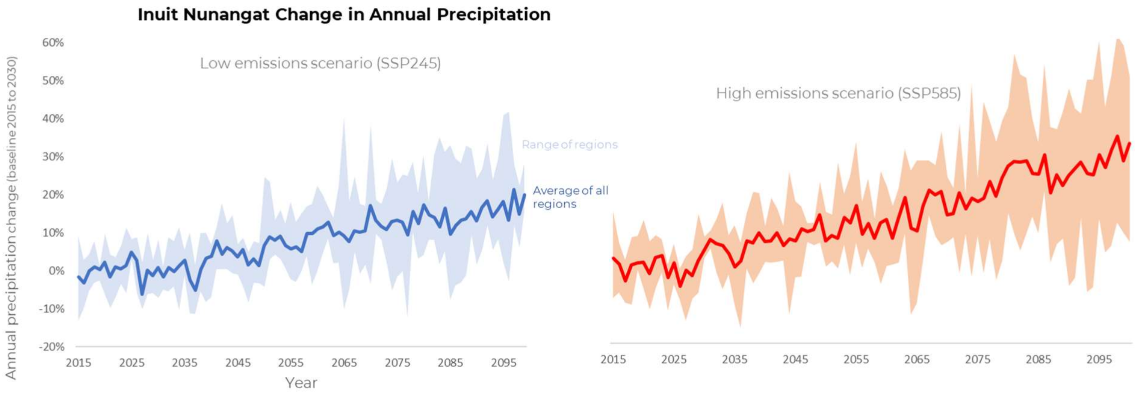

Visibility data

Since global climate models do not presently project ground-level visibility, timeseries were developed by randomly sampling from historic visibility data (see methods). Because of the method, there was no statistically significant change over time in visibility for any region and there were no differences in visibility data between GCMs or emissions scenarios.

The average annual visibility for Inuit Nunangat in the 2020s was 13.57km in our data and was projected to be 13.59km in 2090. Visibility differs across Inuit Nunangat – historically and subsequently in our synthetic timeseries. Based on data available from the past 15 years, average visibility in Nunatsiavut has been the lowest in Inuit Nunangat at 12.76km, and visibility has been the highest in Nunavik at 13.95km. Visibility was the second least common climate or ice variable to cause a model 'fail' across Inuit Nunangat.

Regional trends

Our models suggest that access to water trails will increase by the largest raw number of days across Ellesmere. We project that currently (2015-2030) there are about 57 days/year where Type 1 users have access to water trails across Ellesmere and that this will increase by 36 days/year under the low emissions scenario (SSP245) and 106 more days/year under the high emissions scenario (SSP585) by the end of the century. In comparison, we find that the number of additional good water trail days in Nunatsiavut will be much smaller. We project that the number of good days for Type 1 users in Nunatsiavut is currently 122 days/year and that by the end of the century there will be an additional 39 days/year under the low emissions scenario and 47 additional days/year under the high emissions scenario.

Changes in access to land-based trails vary across Inuit Nunangat as well, with losses in access projected at higher latitudes and gains projected at lower latitudes. Our models suggest the decrease in land access will be across Baffin with a loss of 21 days/year under the low emissions scenario and a loss of 15 days/year under the high emissions scenario. At middle latitudes (ISR and Kitikmeot), our model projects gains in land access under the low emissions scenario and losses under the high emissions scenario. Southern regions (Kivalliq, Nunatsiavut, and Nunavik) are projected to gain land access, although gains are generally small (less than 20 days/year), with the exception of Nunatsiavut under the high emissions scenario (an additional 52 good land days/year by end of century).

Figure S4: *Projected percent of good days per year for each trail type for SSP245 and 585.*

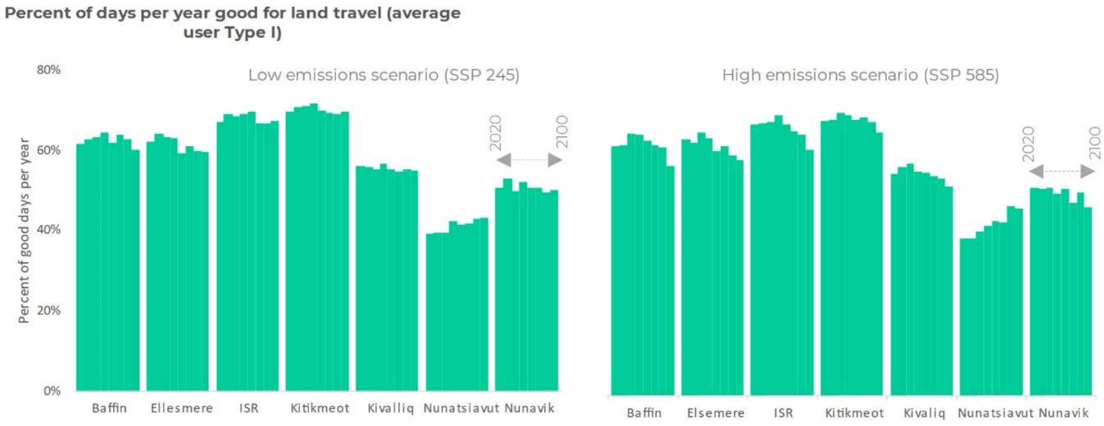

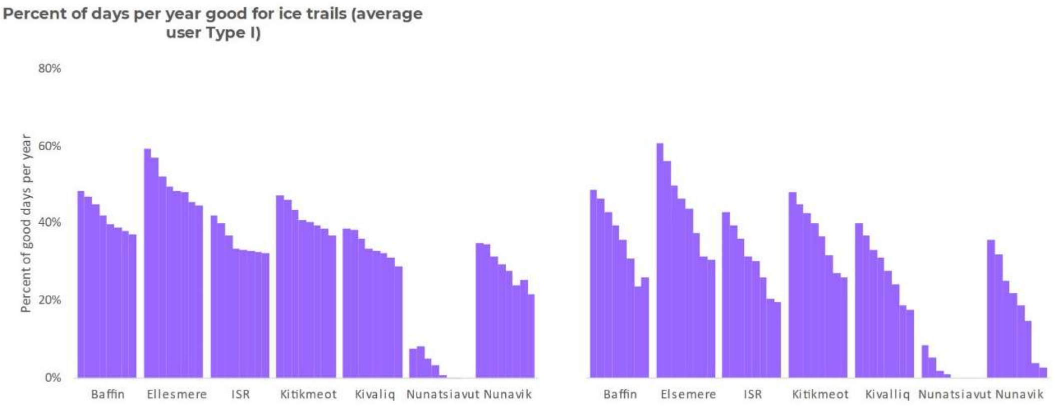

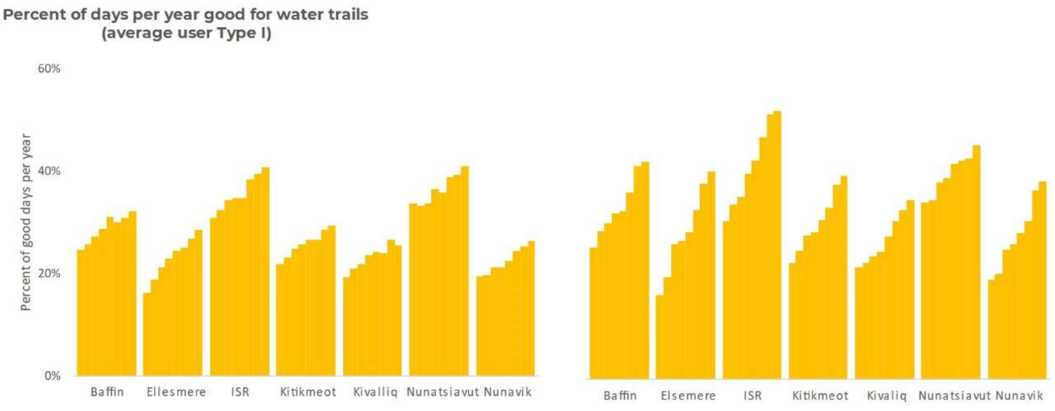

Additional trends by user type

The rate of change in water trail access is highly influenced by user type. Our models project that the quickest rate of change will be for Type 2 users, when averaged across Inuit Nunangat. We project that Type 2 users have access to water trails 13 days/year currently and that the number of days will increase by 4 days/year by the end of the century under the low emissions scenario and 16

days/year under the high emissions scenario. This reflects an increase of 31% and 126%, respectively. On the other hand, we project that there are currently about 165 days/year that water trails are good for Type 3 users across Inuit Nunangat and that the number of good days will increase by 27% (45 days/year) and 76% (125 days/year) for low and high emissions scenarios, respectively.

For land use we see that Type 2 users may benefit most from climate change, with a 14-40% increase projected – up to 147 days/year of increased access in the high emissions scenario by 2100. We project that Type 1 and 3 users will see a change of trail access of less than 2%, with between 214 and 314 days/year projected by 2100 respectively in the high emissions scenario.

Difference by climate model (GCMs):

We used five different global climate models (GCMs) in this study. These GCMs were selected because at the time of analysis, they were the only GCMs that offered all the required variables for the analysis under CMIP6. The GCMs are BCC_CSM2-MR; CMCC-ESM2; MRI-ESM2-0; CCMC_CM2-SR5; and NorESM2-MM.

Results presented in the main manuscript describe data from the ensemble mean of all five GCMs. However, there is significant variation among the GCMs – reflecting a dimension of uncertainty that is important to account for. In Supplementary Table S5 we briefly describe the results for each GCM. All data are the average for Inuit Nunangat. For full details, the datasets generated are available at: https://climatechoices.shinyapps.io/ArcticTrack/

Supplementary Table S5: Differences in projections between different global climate models used in this study

Ensemble:

		**2020 average (2020-2029)**	**Low emissions (2090-2100)**	**High emissions (2090-2100)**
**Climate and ice projections**	Average annual temperature (°C)	-7.99	-7.79	0.48
	Average daily precipitation (cm)	0.01	0.01	0.01
	Average annual wind speed (km/hr)	17.21	17.27	18.03
	Average annual sea ice concentration (% area)	47.02	47.29	20.39
	Average annual sea ice thickness (m)	0.87	0.86	0.29
**Trail projections**	Good land trail days (Type 1 user) per year	212	210	200
	Good ice trail days (Type 1 user) per year	145	146	63
	Good water trail days (Type 1 user) per year	87	86	147

BCC_CSM2-MR:

		**2020 average (2020-2029)**	**Low emissions (2090-2100)**	**High emissions (2090-2100)**
**Climate and ice projections**	Average annual temperature (°C)	-8.70	-5.55	-1.89
	Average daily precipitation (cm)	0.13	0.15	0.16
	Average annual wind speed (km/hr)	18.20	19.00	20.13
	Average annual sea ice concentration (% area)	51.30	40.82	26.90
	Average annual sea ice thickness (m)	0.49	0.33	0.17
**Trail projections**	Good land trail days (Type 1 user) per year	162	169	163
	Good ice trail days (Type 1 user) per year	143	110	59
	Good water trail days (Type 1 user) per year	49	64	83

CMCC-ESM2:

		**2020 average (2020-2029)**	**Low emissions (2090-2100)**	**High emissions (2090-2100)**
**Climate and ice projections**	Average annual temperature (°C)	-7.30	-0.35	3.08
	Average daily precipitation (cm)	0.13	-0.17	0.19
	Average annual wind speed (km/hr)	15.18	15.42	15.81
	Average annual sea ice concentration (% area)	43.30	16.25	2.96
	Average annual sea ice thickness (m)	0.90	0.42	0.32
**Trail projections**	Good land trail days (Type 1 user) per year	222	224	206
	Good ice trail days (Type 1 user) per year	132	46	4
	Good water trail days (Type 1 user) per year	106	156	198

MRI-ESM2-0:

		**2020 average (2020-2029)**	**Low emissions (2090-2100)**	**High emissions (2090-2100)**
**Climate and ice projections**	Average annual temperature (°C)	-6.30	-3.3	0.35
	Average daily precipitation (cm)	0.15	0.16	0.18
	Average annual wind speed (km/hr)	22.93	23.06	23.05
	Average annual sea ice concentration (% area)	47.82	37.40	21.65
	Average annual sea ice thickness (m)	0.91	0.61	0.31
**Trail projections**	Good land trail days (Type 1 user) per year	131.67	139.56	149.82
	Good ice trail days (Type 1 user) per year	118.75	90.08	46.13
	Good water trail days (Type 1 user) per year	52.74	68.31	86.72

CCMC_CM2-SR5:

		**2020 average (2020-2029)**	**Low emissions (2090-2100)**	**High emissions (2090-2100)**
**Climate and ice projections**	Average annual temperature (°C)	-6.57	0.30	3.97
	Average daily precipitation (cm)	0.14	0.18	0.19
	Average annual wind speed (km/hr)	15.25	15.49	15.78
	Average annual sea ice concentration (% area)	41.48	14.60	2.27
	Average annual sea ice thickness (m)	0.82	0.39	0.28
**Trail projections**	Good land trail days (Type 1 user) per year	230	231	214
	Good ice trail days (Type 1 user) per year	126	38	2
	Good water trail days (Type 1 user) per year	108	157	202

NorESM2-MM:

		2020 average (2020-2029)	Low emissions (2090-2100)	High emissions (2090-2100)
**Climate and ice projections**	Average annual temperature (°C)	-11.49	-8.37	-4.26
	Average daily precipitation (cm)	0.12	0.14	0.15
	Average annual wind speed (km/hr)	18.80	18.82	19.31
	Average annual sea ice concentration (% area)	61.25	51.75	37.96
	Average annual sea ice thickness (m)	1.80	1.31	0.97
**Trail projections**	Good land trail days (Type 1 user) per year	160	167	168
	Good ice trail days (Type 1 user) per year	193	159	110
	Good water trail days (Type 1 user) per year	35	58	85

Supplementary References

1. Ford JD, Clarke D, Pearce T, Berrang-Ford L, Copland L, Dawson J, et al. Changing access to ice, land and water in Arctic communities. Nature Climate Change. 2019;9(4):335-+.
2. Clark, D. G. et al. The role of environmental factors in search and rescue incidents in Nunavut, Canada. Public Health 44-49 (2016).
3. Clark, D. G., Ford, J. D., Pearce, T. & Berrang-Ford, L. Vulnerability to injuries associated with land-use activities in Nunavut, Canada. Social science & medicine 169, 18-26 (2016).
4. Laidler, G. J., Elee, P., Ikummaq, T., Joamie, E. & Aporta, C. in SIKU: Knowing Our Ice 45-80 (Springer, 2010).
5. Gearheard, S., Pocernich, M., Stewart, R., Sanguya, J. & Huntington, H. P. Linking Inuit knowledge and meteorological station observations to understand changing wind patterns at Clyde River, Nunavut. Climatic Change 100, 267-294 (2010).
6. Ford, J. D. et al. The Dynamic Multiscale Nature of Climate Change Vulnerability: An Inuit Harvesting Example. Annals of the Association of American Geographers 103, 1193-1211, doi:10.1080/00045608.2013.776880 (2013).
7. Kapsch, M.-L., Eicken, H. & Robards, M. in SIKU: Knowing Our Ice, 115-144 (Springer, 2010).
8. Druckenmiller, M. L., Eicken, H., George, J. C. C. & Brower, L. Trails to the whale: Reflections of change and choice on an Inupiat icescape at Barrow, Alaska. Polar Geography 36, 5-29, doi:10.1080/1088937X.2012.724459 (2013).
9. Gearheard, S. et al. “It's not that simple”: a collaborative comparison of sea ice environments, their uses, observed changes, and adaptations in barrow, Alaska, USA, and Clyde River, Nunavut, Canada. AMBIO: A Journal of the Human Environment 35, 203- 211 (2006).
10. Laidler, G. J. et al. Travelling and hunting in a changing Arctic: assessing Inuit vulnerability to sea ice change in Igloolik, Nunavut. Climatic Change 94, 363-397 (2009).

11. Hansen, W. D., Brinkman, T. J., Leonawicz, M., Chapin, F. S., III & Kofinas, G. P. Changing Daily Wind Speeds on Alaska's North Slope: Implications for Rural Hunting Opportunities. Arctic 66, 448-458 (2013).
12. Laidler, G. J., Dialla, A. & Joamie, E. Human geographies of sea ice: Freeze/thaw processes around Pangnirtung, Nunavut, Canada. Polar Record 44, 335-361 (2008).
13. Aporta, C. Life on the ice: understanding the codes of a changing environment. Polar Record 38, 341-354 (2002).
14. Huntington, H. P. et al. Sea ice is our beautiful garden: indigenous perspectives on sea ice in the Arctic. Sea Ice, 583-599 (2017).
