# Supplementary material for: Projected decrease in trail access in the Arctic
Source: Commun Earth Environ. 2023 Feb 3;4(1):23. doi: 10.1038/s43247-023-00685-w (PMC11041733; doi:10.1038/s43247-023-00685-w)
Supplement: Supplementary file 3 — Reporting Summary [file 43247_2023_685_MOESM3_ESM.pdf]

nature portfolio

Corresponding author(s): Dr. James D. Ford
Last updated by author(s): Jan 8, 2023

Reporting Summary

Nature Portfolio wishes to improve the reproducibility of the work that we publish. This form provides structure for consistency and transparency in reporting. For further information on Nature Portfolio policies, see our Editorial Policies and the Editorial Policy Checklist.

Statistics

For all statistical analyses, confirm that the following items are present in the figure legend, table legend, main text, or Methods section.

n/a	Confirmed	
☐	☒	The exact sample size (n) for each experimental group/condition, given as a discrete number and unit of measurement
☐	☒	A statement on whether measurements were taken from distinct samples or whether the same sample was measured repeatedly
☒	☐	The statistical test(s) used AND whether they are one- or two-sided<br>*Only common tests should be described solely by name; describe more complex techniques in the Methods section.*
☒	☐	A description of all covariates tested
☒	☐	A description of any assumptions or corrections, such as tests of normality and adjustment for multiple comparisons
☐	☒	A full description of the statistical parameters including central tendency (e.g. means) or other basic estimates (e.g. regression coefficient) AND variation (e.g. standard deviation) or associated estimates of uncertainty (e.g. confidence intervals)
☒	☐	For null hypothesis testing, the test statistic (e.g. F, t, r) with confidence intervals, effect sizes, degrees of freedom and P value noted<br>*Give P values as exact values whenever suitable.*
☒	☐	For Bayesian analysis, information on the choice of priors and Markov chain Monte Carlo settings
☒	☐	For hierarchical and complex designs, identification of the appropriate level for tests and full reporting of outcomes
☒	☐	Estimates of effect sizes (e.g. Cohen's d, Pearson's r), indicating how they were calculated

Our web collection on statistics for biologists contains articles on many of the points above.

Software and code

Policy information about availability of computer code

Data collection	All data used in this study is publicly available. Climate data (CMIP6 daily GCM projections) were obtained from https://cds.climate.copernicus.eu/ Visibility data was obtained through Environment Canada historic weather almanac. Data was processed and analyzed using RCran, RStudio, and ArcGIS software.
Data analysis	Code used for data cleaning, aggregation, and analysis is available through GitHub: https://github.com/dylangclark/ArcticTrack<br>Results can also be viewed on a ShinyApp: https://climatechoices.shinyapps.io/ArcticTrack/

For manuscripts utilizing custom algorithms or software that are central to the research but not yet described in published literature, software must be made available to editors and reviewers. We strongly encourage code deposition in a community repository (e.g. GitHub). See the Nature Portfolio guidelines for submitting code & software for further information.

March 2021

Data

Policy information about availability of data

All manuscripts must include a data availability statement. This statement should provide the following information, where applicable:

- Accession codes, unique identifiers, or web links for publicly available datasets
- A description of any restrictions on data availability
- For clinical datasets or third party data, please ensure that the statement adheres to our policy

The datasets generated and analyzed during the current study are available from the corresponding author on reasonable request. Aggregated data may be viewed and downloaded through the ShinyApp: https://climatechoices.shinyapps.io/ArcticTrack/

Human research participants

Policy information about studies involving human research participants and Sex and Gender in Research.

Reporting on sex and gender	*Use the terms sex (biological attribute) and gender (shaped by social and cultural circumstances) carefully in order to avoid confusing both terms. Indicate if findings apply to only one sex or gender; describe whether sex and gender were considered in study design whether sex and/or gender was determined based on self-reporting or assigned and methods used. Provide in the source data disaggregated sex and gender data where this information has been collected, and consent has been obtained for sharing of individual-level data; provide overall numbers in this Reporting Summary. Please state if this information has not been collected. Report sex- and gender-based analyses where performed, justify reasons for lack of sex- and gender-based analysis.*
Population characteristics	*Describe the covariate-relevant population characteristics of the human research participants (e.g. age, genotypic information, past and current diagnosis and treatment categories). If you filled out the behavioural & social sciences study design questions and have nothing to add here, write "See above."*
Recruitment	*Describe how participants were recruited. Outline any potential self-selection bias or other biases that may be present and how these are likely to impact results.*
Ethics oversight	*Identify the organization(s) that approved the study protocol.*

Note that full information on the approval of the study protocol must also be provided in the manuscript.

Field-specific reporting

Please select the one below that is the best fit for your research. If you are not sure, read the appropriate sections before making your selection.

☐ Life sciences ☒ Behavioural & social sciences ☐ Ecological, evolutionary & environmental sciences

For a reference copy of the document with all sections, see nature.com/documents/nr-reporting-summary-flat.pdf

Behavioural & social sciences study design

All studies must disclose on these points even when the disclosure is negative.

Study description	Data analyzed in this study are quantitative. The study relies on climate projections and historic climate data. All datasets are publicly available. Data is analyzed based on thresholds established in previously published works.
Research sample	the study focuses on all Inuit Nunangat
Sampling strategy	Climate projections were obtained for 100km radius zones surrounding all 53 communities across Inuit Nunangat (in the Canadian Arctic). Results are presented in regional trends in order to account for potential variation at the community spatial scale. This is common among other studies that apply climate models across the Canadian Arctic.
Data collection	Data were collected use RCran pipes to climate model ftp repositories and through historic weather databases. All code used to pull, clean, aggregate, and analyze the climate and weather data are available on GitHub.
Timing	All data were collected between November, 2021 and February, 2022.
Data exclusions	Missing historic visibility data were excluded from the timeseries used to create synthetic visibility data. Exclusion criteria were established prior to the data cleaning and analysis. Any year with more than 4 missing days was excluded. This decision was made in order to maintain precise seasonal distributions of visibility. Because visibility data was intended to be stationary, we were able to confirm that the exclusions did not alter the annual or seasonal distributions.

Non-participation	This is not applicable
Randomization	This is not applicable

Reporting for specific materials, systems and methods

We require information from authors about some types of materials, experimental systems and methods used in many studies. Here, indicate whether each material, system or method listed is relevant to your study. If you are not sure if a list item applies to your research, read the appropriate section before selecting a response.

Materials & experimental systems

n/a	Involved in the study
☒	☐ Antibodies
☒	☐ Eukaryotic cell lines
☒	☐ Palaeontology and archaeology
☒	☐ Animals and other organisms
☒	☐ Clinical data
☒	☐ Dual use research of concern

Methods

n/a	Involved in the study
☒	☐ ChIP-seq
☒	☐ Flow cytometry
☒	☐ MRI-based neuroimaging
